# SG-APSIC1180: Successful reduction in the number of hospital-acquired dialysis- catheter–related bloodstream infections: Quality improvement initiative

**DOI:** 10.1017/ash.2023.62

**Published:** 2023-03-16

**Authors:** Sreekanth Koduri, Tan Seow Yen, Prasanna Thirukonda, Maria Theresa, Alvin Chew Zhen Jie, Wang Hwee May, Jane Caroline Van Der Straaten

**Affiliations:** Changi General Hospital, Singapore; Changi General Hospital, Singapore; Changi General Hospital, Singapore; Changi General Hospital, Singapore; Changi General Hospital, Singapore; Changi General Hospital, Singapore; Changi General Hospital, Singapore

## Abstract

**Objectives:** Patients undergoing hemodialysis using a catheter are at significant risk of developing central venous catheter–related bloodstream infections (CRBSIs), especially with methicillin-resistant *Staphylococcus aureus* (MRSA), resulting in increased morbidity, mortality, and cost. In our 1,000-bed regional hospital, the average CRBSI (any bacteria) rate in patients dialyzing via dialysis catheters was 1.44 per 1,000 catheter days, and the average CRBSI (MRSA) rate was 0.56 per 1,000 catheter days. A quality improvement project was initiated to reduce the overall dialysis CRBSI and CRBSI-MRSA by 50%. **Methods:** Following the formation of a multidisciplinary team, the catheter-insertion protocols and catheter-care protocols were standardized throughout the hospital. We adopted a well-established scientific quality improvement method, plan–do–study–act (PDSA) cycle model for all interventions that were implemented. The patients and general ward nursing staff were provided education and training in dialysis catheter care. **Results:** The project was initiated in January 2016, and the initial improvement was seen from July 2017 onward. Analysis of the data since 2016 showed a steady improvement in the overall CRBSI rates, as well as CRBSI-MRSA rates. The average CRBSI rate improved to 0.76 per 1,000 catheter days, and the average CRBSI-MRSA rates improved to 0.15 per 100 catheter days in the calendar year 2021. **Conclusions:** Because the causes of these infections are multifactorial, emphasis should be placed on improving care processes from the patient preparation phase prior to catheter insertion to regular catheter care in the inpatient wards and dialysis units. We attribute the success of our project to involving all stakeholders and obtaining constant feedback from the staff. We successfully applied PDSA cycles to make relevant incremental changes.

